# The relationship between loneliness, depression and cognition: Evidence from smartphone‐based cross‐sectional and ecological momentary assessment

**DOI:** 10.1002/alz.089194

**Published:** 2025-01-03

**Authors:** Sol Fittipaldi, Vanessa Teckentrup, Anna M Rosická, Brian Lawlor, Claire M Gillan

**Affiliations:** ^1^ Latin American Brain Health Institute (BrainLat), Universidad Adolfo Ibañez, Santiago Chile; ^2^ Global Brain Health Institute, Trinity College Dublin, Dublin Ireland; ^3^ Trinity College Dublin, Dublin Ireland

## Abstract

**Background:**

Loneliness is associated with lower cognitive function and may increase dementia risk. However, it is unclear if this effect is mediated by depression. Resolving this issue is important to design effective interventions to promote healthy aging. We adopted a complementary between‐ and within‐person approach, which allowed us to study cross‐sectional relationships as well as the dynamic interactions between loneliness, mood, and cognition in natural environments over time.

**Method:**

A total of 3,416 participants between 18 and 84 years (1,149 male; M age = 45.89±14.55) completed cross‐sectional self‐reported questionnaires of loneliness and depression alongside gamified assessments of memory, processing speed, cognitive flexibility, and planning through a smartphone app, Neureka. A subsample of 286 participants between 18 and 82 years (89 male; M age = 50.14±13.16) also underwent 8‐week ecological momentary assessment (EMA) reporting every 12 hours how lonely and down they felt. We measured cognition at these same timepoints using a recently validated passive measure of cognitive processing speed (digital questionnaire response time, DQRT). Multiple regressions and network analysis were performed to analyze cross‐sectional and EMA data, respectively.

**Result:**

Loneliness and lower cognitive function were associated cross‐sectionally (all ps<.001) except for planning (p = .08). Significant effects did not survive after controlling for depression (all ps>.06). Turning to EMA data, a contemporaneous network analysis showed that within‐person 12‐hour fluctuations in loneliness were related to fluctuations in mood (r = .35, p<.001). However, fluctuations in mood (but not loneliness) were linked to changes in DQRT (r = .13, p<.001). To understand the causal path, we conducted temporal network analysis, which revealed a bi‐directional relationship between loneliness and low mood (β = .08 and β = .06, respectively, both p<.001). In contrast, lower mood predicted slower DQRT 12‐hours later (β = .02, p = 0.03) and not the other way around (β = .005, p = 0.43). In older adults, loneliness and low mood were less related to one‐another, but the relationship between low mood and slower DQRT was stronger.

**Conclusion:**

Depression symptoms mediate the effect of loneliness on cognition, both cross‐sectionally and when assessed within‐person. Older participants show less coupling between loneliness and low mood, but stronger coupling between low mood and slower cognition. Results have implications for differential interventions across the lifespan.

